# Defect repair in rat mandible with hydroxyapatite cement comparad to small intestine submucosa

**DOI:** 10.1016/S1808-8694(15)30055-0

**Published:** 2015-10-19

**Authors:** Andréa Thomaz Soccol, Silvio Bettega, Lúcia Noronha, Sheila Sass, Vanete T. Soccol, Marcos Renato Scholz, Marcos Mocellin

**Affiliations:** a Medical Student of the 6th year. Otorhinolaryngology resident - University Hospital of Paraná.; b PhD in Surgery, Otorhinolaryngology, Professor at the Federal University of Paraná.; c PhD, Assistant Professor of Pathology - UFPR.; d Medical Student of the 6th year of the Pontifícia Universidade Católica do Paraná.; e PhD, Assistant Professor of Basic Pathology - UFPR.; f MS student in Biotechnology - Federal University of Paraná. MD, Orthopedist.; g PhD, Full Professor of Otorhinolaryngology - UFPR. Head of the Otolaryngology Department - University Hospital - Federal University of Paraná. Universidade Federal do Paraná.

**Keywords:** defect repair, hydroxyapatite, small intestine submucosa

## Abstract

**Aim:** The aim of this study was to evaluate the bone formation in surgically created defects of rabbit mandibles by synthetic hydroxyapatite of calcium compared to small Intestine Submucosa. **Material and Method:** 24 mices lineage Wisthar-Furth were used. A bony defect of 0,75 cm x 1,5 cm in mandibular ramus was accomplished in all animals. The hydroxyapatite implants were placed on the left hemimandiblein groupI, small Intestine submucosa in group II, and the right served as control. The euthanasia was accomplished in the 40° postoperative day, it was proceeded the macroscopic and histological analysis. **Results:** medium length in millimeters of the hemimandibless in the hydroxyapatite group was of 3,75, in the small intestine submucosa 3,03 and the control group was of 2,63 (p: 0,022). Histomorphometry study reaveled new bone grown in 76,64% of the total area in hydroxyapatite group (p: 0,022). In Small Intestinal submucosa group new bone grown in 63,64% do total (p: 0,0022). **Discussion:** satisfactory bone integration was observed of the synthetic hydroxyapatite in that experimental model. Small intestinal submucosa cause osteoinduction **Conclusion:** using hydroxyapatite of calcium resulted in formation of significantly larger volume frations of new bone when compared to small intestinal submucosa group.

## INTRODUCTION

Many biomaterials are being tested because they can cause bone regeneration, such as: calcium hydroxyapatite and tricalcium phosphate^1^. All these porous synthetic surrogates share advantages in relation to auto and allografts, including their easy sterilization and storage, and their unlimited availability. Among their disadvantages we have: delicate handling, variable degrees of resorption, poor performance in diaphysis defects and potential adverse effects on bone remodeling^2^.

Synthetic hydroxyapatite, [CaIO(PO4)(OH)], is an inorganic material commonly used in bone gaps and as a constituent in the mineral phase of calcified tissues. It is biocompatible and bears osteoconduction, and this makes it the most important bone surrogate of modern times; because it is used in bone defects without load or in gaps in which loads, tortional stresses or shearing forces are neutralized by rigid implants such as plates and screws^3^.

Bone regeneration promoted by hydroxyapatite has been studied in different animal and human models. Its first implants in animal models were made in proximal tibial defects in dogs. A fast graft incorporation was seen after graft implant, no adverse effects, bringing about high bone regeneration^4^.

The porcine Small Intestine Submucosa (SIS) is a multilaminar acellular layer made up mostly of collagen that has shown to have characteristics that make it a proper material for tissue bioengineering in different anatomical sites^5^.

According to many authors, SIS has the advantage of being made up of 90% type I collagen, fibronectines, growth factors, glycosaminoglycans, protaminoglycans and glycoproteins^6^. Since it is an acellular tissue, fribonectine is the element responsible for inducing local celularity^7^.

As to the immune reactions triggered by porcine SIS, Metzger et al. concluded that this membrane induces an immune response in rats, activating T-helper-2 cells^8^. There was also a drop in the levels of inflammatory cytokines, as well as of alpha tumoral necrosis factor, IL I and IL VI. The authors concluded that porcine SIS does not promote rejection reactions when implanted.

Hodde et al. advocate that this capacity is due to the presence of glycosaminoglycans in SIS, which are able to activate numerous cytokines and growth factors that participate in healing and revascularization processes, thanks to the presence of vascular endothelial growth factor (VEGF), which is able to induce the formation of similar structures, and fenestrated capillaries in the fibrin matrix^9^.

The objective of the present study is to evaluate bone regeneration in a bone defect created in rat mandibles, comparing two biomaterials, synthetic calcium hydroxyapatite and porcine small intestine submucosa.

## MATERIALS AND METHODS

In performing this study we respected the rules of the Brazilian College of Animal experimentation (COBEA). We used 24 adult rats from the Wisthar-Furth lineage, weighing between 180 and 220 grams. They were kept in proper environment under natural lighting and temperature, and fed water and ration ad libitum.

The animals were anesthetized via intramuscular injection of Ketamine 40 mg/kg, Diazepam 2 mg/kg and Butorphanol 2 mg/kg. A 3cm incision was made in order to expose the mandible. A 0.75cm x 1.5cm bone defect was created on the body of each mandible in all animals using a low RPM Sorensen® #7.5 spherical burr.

The animals were divided in two groups: group I received filling with caustic hydroxyapatite in the left mandible and group II had the left side bone defect filled with porcine small intestine submucosa.

Prophylaxis was carried out with antibiotics during the procedure with cephazolin 0.01 ml/kg. Such animals remained in single cages, with controlled lighting and ad libitum diet. The animals were slaughtered on the 40th day of postop.

Macroscopic analysis was carried out through measuring the diameter of bone calluses (growth). For microscopic analysis the specimens were sent to be fixed in paraffin and were later processed according to conventional hystology techniques, and later on they were dyed with hematoxylin-eosin. For morphometric assessment we carried out 6 measurements in 6 different microscopic fields. This evaluation was carried out using the Image Pro-Plus software, coupled to a Sony video camera and a BX 50 microscope, calibrating the magnification for a 10x lens, using the area morphometric application through a difference in color between bone tissue and the connective tissue highlighted by dyeing. We assessed bone neoformation, area porosity, amount of mature bone and immature bone.

For statistical analysis of the macroscopic varieties (diameter), we used Wilcoxon’s non-parametric test. And for histology analysis (porosity percentage, percentage of new bone formed, percentage of mature bone, percentage of immature bone) we used the binomial test. We set in 5% (p< 0.05) the level of rejection for the null hypothesis.

## RESULTS

As for the clinical analysis, all the animals remained healthy and did not have complications in their post-operative outcome, except for one animal from Group I and two animals from Group II that had bone infection and were removed from the study.

The macroscopic analysis showed a greater formation of bone callous in the group that received hydroxyapatite, when compared to the group that received small intestine submucosa. The average length, in millimeters, of the hemi mandibles of the hydroxyapatite group was 3.75; in the small intestine submucosa group was 3.03; and the control group had 2.63 (p: 0.0022) in length. Groups I and II had better results when compared to the control group, without the use of any graft.

Area morphometry showed that the control group (without any graft) had bone neoformation in 28.40% and porosity remained in 71.59%.

The group with hydroxyapatite had bone area neoformation corresponding to 76.64% of the total (p: 0.0022). Of this neoformed bone, 83.77% already was mature bone. In this same evolutionary stage, the control group showed only 24.88% (Figure 2a, Figure 2b).

**Figure 2a:**
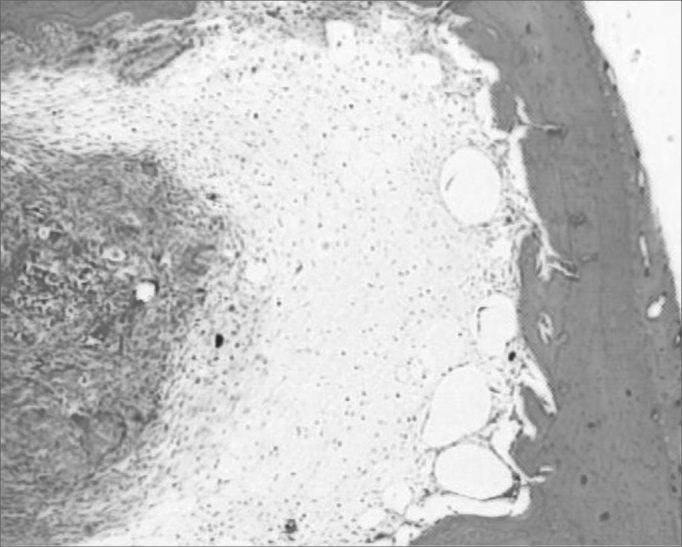
Hematoxylin and eosin slide in a specimen from the control group.

**Figure 2b:**
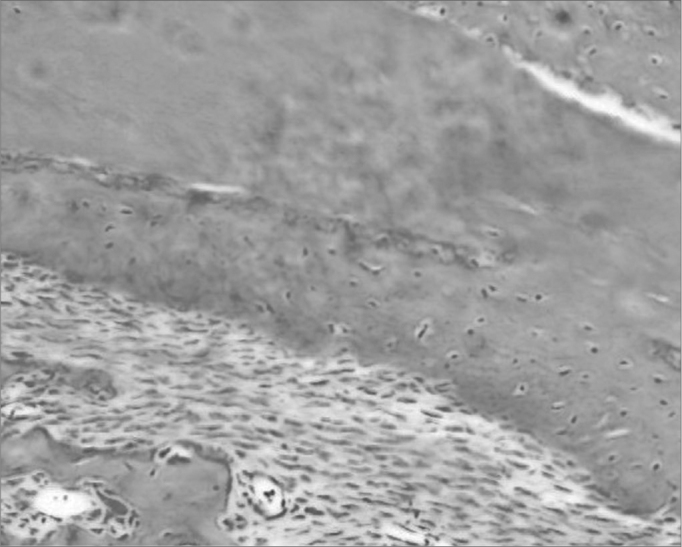
Slide dyed with HE from the hydroxyapatite group - We see bone neoformation.

In the group with porcine SIS, bone neoformation added up to a corresponding area of 63.64% of the total (p: 0.0022). Of this neoformed bone, 63.77% already was mature bone at in this same evolutionary stage, the control group had only 36.23% (p: 0.0025).

## DISCUSSION

Recently, literature has shown promising results as to the use of SIS grafts in different anatomical structures, such as the urinary bladder, urethra, tendons, esophagus, arteries, veins and abdominal wall^10^, ^11^.

After implantation there is a fast neovascularization, infiltration and spatial organization. This may be seen in Sanduski’s study, in which seven days after SIS implant in canine carotid arteries the observed a fibrin coating and neointima in the graft, and in the 90th day of post-op, the SIS implant site was equal to that of a normal artery^12^, ^13^.

About immune reactions triggered by porcine SIS, Metzger et al. (1997) concluded that this membrane induces immune response and activates T-helper 2 cells in rats and they also saw a reduction in the levels of inflammatory cytokines, as well as alpha tumoral necrosis factor an IL 1 and 6. The authors concluded that porcine SIS does not promote rejection reaction when implanted^7^.

Badylak el al. (1995), in an experimental study in which they caused an intentional infection by Staphylococcus aureus, in order to compare SIS with polyetrafluoretilene, and had 56% of infection rate in synthetic prosthesis and 0% in porcine SIS^15^, ^16^, ^17^.

According to Hodde et al., the porcine small intestine submucosa (SIS) induces bone conduction and thus facilitates the filling up of a bone defect that was been surgically created. It acts as an optimal repair material and induces medullar cells growth in the graft bone neoformation, thus promoting a fast cartilage formation as well^9^.

Hydroxyapatite has been approved for human use by the American Food and Drug Administration (FDA) only for metaphysis traumatic defects. However, it has been used in daily medical practice in a number of situations^18^.

Bone graft incorporation happens in 5 stages: 1) inflammatory - causing an inflammatory response in the host; 2) tissue revascularization; 3) bone conduction, in which the graft functions as a framework for the growth of vessels and bone formation; 4) osteoinduction, in which host mesenchimal cells are induced by proteins (BMP) found in the graft and change into osteoblasts, and 5) bone remodeling with characteristics of continuous bone formation and resorption^5^.

Kühne et al. made a radiological and histological assessment of coral porous hydroxyapatite in an experimental study in rabbits. The implants were carried out in their femoral condiles in order to repair empty cavities, thus proving bone integration when porous hydroxyapatite is used^19^.

Uchida et al. carried out an experimental study in rabbits by implanting sea coral duplicated ceramics in standard bone defects, proving bone graft integration. In the present study we saw a greater formation of a macroscopic bone callous in the hydroxyapatite group, when we could observe the graft-implant integration^20^.

Bucholz et al. compared the coral porous hydroxyapatite applicability or autogenous bone graft for the treatment of tibial fractures in humans, and did not find significant differences between the two groups, proving the possibility of using hydroxyapatite in bone defects^2^. Yamamoto et al. used hydroxyapatite to fill up defects after the excision of benign bone tumors and achieved total radiographic graft incorporation 3 months after its implant^21^.

Karabatsos et al. implanted hydroxyapatite in the femur of a canine model and obtained significant bone integration in the graft-implant interface^4^.

The microscopic analysis tried to assess the morphometry of the region where the bone defect was created, observing an abundant bone neoformation in 76.64% of the hemi mandibles in which hydroxyapatite was used, as well as an advanced level of bone maturation, since 83.77% of the neoformed bone bore mature bone characteristics. Porcine SIS also proved to be a good osteoconductor; however the level of bone neoformation was greater in the hydroxyapatite group.

We could see satisfactory porous hydroxyapatite integration to the mandible bone in this experimental model. Macroscopic, radiologic and microscopic results were better when this type of graft was used when compared to the group that received submucosa. However, both grafts had bone induction results which were clearly better when compared to the control group.

Future experiments in this same line of research must be implemented, using other animal models that allow the creation of an anatomical larger bone defect and that can have a longer post-surgery time.

**Figure 1a:**
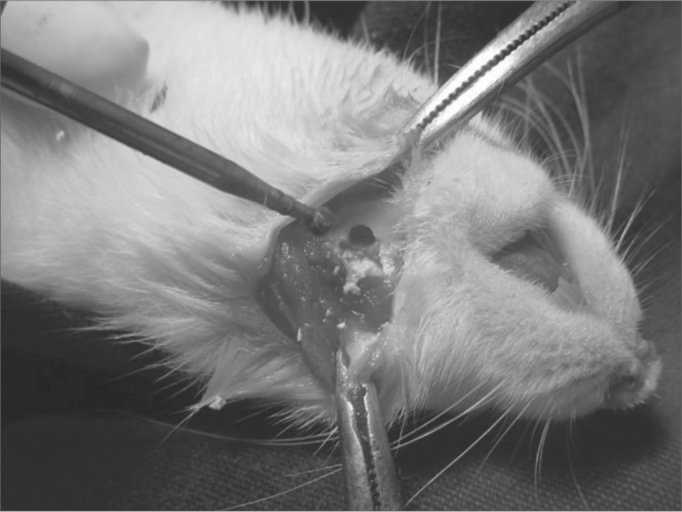
Perforation in the rat mandible - We see the mandible perforation with the low RPM 7.5 Sorense burr.

**Figure 1b:**
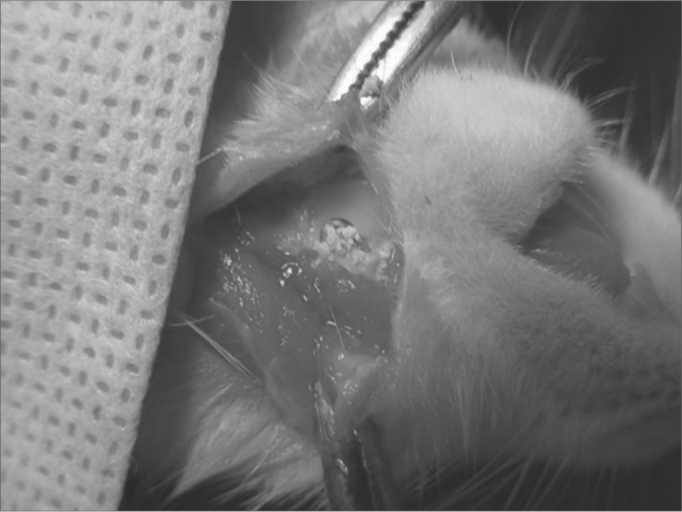
Filling the bone defect created with caustic hydroxyapatite.

## CONCLUSION

SIS seemed to make it easier to fill up the surgical defect created in rat mandibles. Further studies are necessary in order to better define the cellular basis for this activity, as well as the assessment of potential SIS applications in grafts to repair bone gaps in human beings.

Coral porous hydroxyapatite integrated to the recipient experimental bone model, allowing early and adequate bone neoformation.

When we compare both biografts, coral hydroxyapatite achieved better results.

## References

[1] Chiroff RT, White EW, Weber JN, Roy DM (1975). Tissue ingrowth of replamineform implants. J Biomed Mater Res Symp.

[2] Bucholz RW, Carlton A, Holmes R (1989). Interporous hydroxyapatiteas a bone graft substitute in tibial plateau fractures. Clin Orthop Rel Res.

[3] Burchardt H (1987). Biology of bone transplantation. Orthop Clin North Am.

[4] Karabatsos B, Myerthall ST, Fornasier V, Maistrelli G (2001). Osseointegration of hydroxyapatite porous-coated femoral implants in a canine model. Clin Orthop Rel Res.

[5] Fry DE, Milholen L, Harbrecht PT (1986). Iatrogenic ureteral injury, options in management. Arch Surg.

[6] Schmidt CE, Baier JM (2000). Acellular vascular tissue: natural biomaterials for tissue repair and tissue engineering. Biomaterials.

[7] McPherson TG, Badylak SF (1998). Characterization of fibronectin derived from porcine small intestinal submucosa. Tissue Engineering.

[8] Murata K, Sekino T, Ikeda T (1995). Glycosaminoglycan components in duodenum with advancing age and in patients with progressive systemic sclerosis. Digestion.

[9] Hodde JP, Badylak SF, Brightman AO (1996). Glycosaminoglycan content of small intestine submucosa: A bioscaffold for tissue replacement. Tissue Engineering.

[10] Owen TJ, Lantz GC, Hiles MC, Martin BR, Geddes LA (1997). Calcification potential of small intestinal submucosa in a rat subcutaneous model. J Surg Res.

[11] Peel SAF, Chen H, Renlund R (1998). Formation of a SIS-cartilage composite graft in vitro and its use in the repair of articular cartilage defects. Tissue Engeneering.

[12] Sandusky GE, Badylak SF, Morff RJ, Johnson WD, Lantz GC (1992). Histologic findings after in vivo placement of small intestine submucosal vascular grafts and saphenous vein grafts in the carotid artery in dogs. Am J Pathol.

[13] Sandusky GE, Lantz GC, Badylak SF (1995). Healing comparison of small intestine submucosa and PTFE grafts in canine carotid artery. J Surg Res.

[15] Bandylak SF, Lantz GC, Coffey A, Geddes LA (1989). Small intestinal submucosa as a large diameter vascular graft in the dog. J Surg Res.

[16] Bandylak SF, Lantz GC, Coffey A, Geddes LA, Tacker WA (1994). Comparison of the resistence to infection of intestinal mucosa arterial autografts versus polytetrafluoretilene arterial protheses in a dog model. J Vasc Surg.

[17] Bandylak SF, Tullis R, Kokini K (1995). The use of xenogeneic small intestine submucosa as a biomaterial for Achilelles tendon repair in a dog model. J Biomed Mater Res.

[18] Hahmer R, Mooney V, Bucholz R, Tencer A (1986). A coralline hidroxyapatite bone graft substitute. Clin Orthop.

[19] Kühne JH, Bartl R, Frisch B, Hammer C, Jansson V, Zimmer M (1994). Bone formation in coralline hydroxyapatite: effects of pore size studied in rabbits. Acta Orthop Scand.

[20] Uchida A, Nade SML, McCartney ER, Ching W (1984). The use of ceramics for bone replacement: a comparative study of three different porous ceramics. J Bone Joint Surg.

[21] Yamamoto T, Onga T, Marui T, Mizuno K (2002). Use of hydroxyapatite to fill cavities after excision of benign bone tumors. J Bone Joint Surg.

